# Role of the posterodorsal medial amygdala in predator odour stress‐induced puberty delay in female rats

**DOI:** 10.1111/jne.12719

**Published:** 2019-04-29

**Authors:** Xiao Feng Li, Daniel A. Adekunbi, Hussah M. Alobaid, Shengyun Li, Michel Pilot, Stafford L. Lightman, Kevin T. O'Byrne

**Affiliations:** ^1^ Department of Women and Children's Health Faculty of Life Sciences and Medicine King's College London London UK; ^2^ Zoology Department College of Science King Saud University Riyadh Saudi Arabia; ^3^ Henry Wellcome Laboratory for Integrative Neuroscience and Endocrinology University of Bristol Bristol UK

**Keywords:** amygdala, CRF, predator, puberty, stress

## Abstract

Puberty onset is influenced by various factors, including psychosocial stress. The present study investigated cat‐odour stress on puberty onset and oestrous cyclicity in rats. Female weanling rats were exposed to either soiled cat litter or fresh unused litter for 10 consecutive days. Following vaginal opening (VO), rats were smeared for 14 days to determine oestrous cyclicity. Anxiety‐like behaviour was assessed using standard anxiety tests. Brains were collected to determine corticotrophin‐releasing factor (CRF), CRF receptor 1 (CRF‐R1) and CRF receptor 2 (CRF‐R2) mRNA in the paraventricular nucleus (PVN), as well as the central nucleus of the amygdala (CEA) and the medial nucleus of the amygdala (MEA). Cat odour delayed VO and first oestrus, disrupted oestrous cycles and caused anxiogenic responses. Cat odour elicited increased CRF mRNA expression in the PVN but not in the CeA. CRF‐R1 and CRF‐R2 mRNA levels in the PVN and CeA were unaffected by cat odour; however, CRF‐R1 mRNA levels were decreased in the MeA. The role of CRF signalling in the MeA, particularly its posterodorsal subnucleus (MePD), with respect to pubertal timing was directly examined by unilateral intra‐MePD administration of CRF (0.2 nmol day^‐1^ for 14 days) via an osmotic mini‐pump from postnatal day 24 and was shown to delay VO and first oestrus. These data suggest that CRF signalling in the MePD may be associated with predator odour‐induced puberty delay.

## 1 INTRODUCTION

The maturation of the hypothalamic‐pituitary gonadal (HPG) axis, which drives the onset of puberty, is influenced by numerous intrinsic and extrinsic factors, including stress, especially psychosocial stress, which can either delay or advance puberty in both humans and experimental animals.^1^, ^2^, ^3^ Previous studies have shown that i.c.v. administration of corticotrophin‐releasing factor (CRF) in prepubertal female rats delays the timing of puberty, whereas CRF antagonism advances puberty onset.^4^ Thus, the stress neuropeptide CRF plays a key role in the maturation of the HPG axis. Although a major site for CRF expression is the hypothalamic paraventricular nucleus (PVN), the central driver of the hypothalamic‐pituitary‐adrenal (HPA) axis, lesion studies suggest that PVN CRF may not be critical in stress‐induced disturbance of the HPG axis.^5^ However, CRF neurones and projections are not limited to the PVN and have a wide distribution in the brain,^6^ including the amygdala. The amygdala is composed of a complex of nuclei including the central nucleus of the amygdala (CEA) and the medial nucleus of the amygdala (MEA). Numerous CRF neurones are found in the CeA and CRF‐receptor (R)1 and CRF‐R2 are located both in the CeA and MeA. Under chronic stress conditions, CRF expression in the amygdala is increased.^7^ There is also considerable evidence to suggest that the amygdala facilitates the stress response.^8^ Besides being involved in the stress response, both CRF and the amygdala are also importantly involved in fear and anxiety.^9^, ^10^ Therefore, CRF in the amygdala may play a role in disorders linked to both excessive stress, fear and anxiety, such as post‐traumatic stress disorder (PTSD).^10^

Chronic stress impairs reproductive function, which is a process that involves the CeA and MeA.^11^ Indeed, there is a differential modulatory role for these two amygdala nuclei in terms of stress‐induced suppression of pulsatile luteinising hormone (LH) secretion, with the MeA being more responsive to psychological stress and the CeA to physiological stress.^11^ Recent *in vivo* electrophysiological studies in rats have demonstrated that the MeA had the highest frequency of response and distinct firing pattern to predator urine compared to other amygdaloid nuclei,^12^ which confirms the importance of this neurocircuitry in psychological stress. Moreover, lenti‐viral overexpression of CRF in the CeA in pre‐pubertal female rats, mimicking the effect of chronic stress, disrupts reproductive function and leads to irregular oestrous cycles, increased anxiety and advanced puberty.^2^, ^13^ It is therefore reasonable to hypothesise that CRF signalling in the amygdala may be a key player in cross‐talk between stress‐induced activation of the HPA axis and suppression of the HPG axis.

The MeA, particularly its posterodorsal subnucleus (MePD) receives olfactory information from environmental and sexual cues,^14^, ^15^ which are relayed to the medial preoptic area (mPOA) where the gonadotrophin‐releasing hormone (GnRH) neurones are located to bring about the appropriate reproductive response. The MePD also undergoes dramatic anatomical changes during puberty 16, 17 and exerts a regulatory role on puberty timing, as indicated by studies in rats showing that MePD lesions caused puberty advancement,^18^ whereas its electrical stimulation delayed puberty onset.^19^ In addition, the MePD has been implicated in ovulation and oestrous cyclicity, as well as gonadotrophic hormone secretion.^18^, ^20^

In the present study, we tested the hypothesis that predator odour‐induced stress would delay puberty timing and alter oestrous cyclicity. We also assessed both social and anxiety‐like behaviour as proxy measures of psychological stress in predator odour‐stressed prepubertal female rats. Finally, we determined the role of CRF signalling in the MeA and, more specifically, the MePD with respect to mediating changes in pubertal timing.

## 2 MATERIALS AND METHODS

### 2.1 Animals, predator odour stress and puberty evaluation

All procedures were conducted in accordance with the United Kingdom Home Office Animals (Scientific Procedures) Act 1986. The protocols were approved by the Committee on the Ethics of Animal Experimentation of King's College London. Late pregnant Sprague‐Dawley rats were supplied by Charles River (Margate, UK) and housed under a 12:12 hours light/dark cycle (lights on 7.00 am) at 22 ± 2°C with food and water available ad lib. Litters were reduced to eight to 12 pups on postnatal day (PND) 1 (birth, day 0). They were weaned on PND 21 and housed in groups of three or four female rats per cage and weighed every 3 days. To determine whether the timing of puberty could be affected by a predator odour stress, female pups were randomly assigned to either a treatment group (n = 12) or control group (n = 10) after weaning. For the predator odour stress (cat odour‐treated), an individual rat was placed in isolator animal cage (37 × 26 × 17 cm) in which a well‐soiled cat litter (in use by the cat for 3 days) was sifted to remove clumps of faeces and placed in a dish (diameter 5.5 cm) in the middle of the cage. The duration of each exposure was 15 min, after which the rat was returned to the home cage, with this procedure being repeated for 10 consecutive days (PND 21‐30) to model the effect of chronic stress. Sham exposures were conducted by placing the rat in an identical cage (used only for the control treatment) but with fresh, unused cat litter for the same amount of time. All exposures took place between 10.00 am and 12.00 pm each day.

From PND 28, the pre‐pubertal female rats were monitored daily for vaginal opening. Once vaginal opening occurred, vaginal smears were taken daily for 14 consecutive days to detect first oestrus as a marker of puberty onset and the stage of the oestrous cycle. Normal oestrous cyclicity was defined as having at least two consecutive normal cycles, which lasts for 4‐5 days with 1‐2 days of oestrus. Cycle length, with three stages observed in the correct order, was determined by the number of days between each occurrence of oestrus.^21^

### 2.2 Tissue collection and reverse transcriptase‐polymerase chain reaction (RT‐PCR)

To examine the effect of predator stress on levels of CRF, CRF receptor 1 (CRF‐R1) and CRF receptor 2 (CRF‐R2) mRNA in the PVN and amygdala (CeA and MeA), predator odour stressed and control animals were killed by decapitation 14 days post vaginal opening. The brains were then removed, snap frozen on dry ice and stored at −80°C until the brains were cut into coronal sections (thickness of 300 μm) using a cryostat (Bright Instrument Co., Luton, UK). Using the micropunch method of Palkovits,^22^ bilateral punches (0.5 mm in diameter) of the CeA (bregma −2.12 to −3.14 mm), MeA (bregma −2.80 to −3.60) and PVN (bregma −0.70 to −2.10 mm) were taken according to the rat brain atlas of Paxinos and Watson.^23^ For confirmation of correct punch position, sections were fixed with formalin, stained in crystal violet solution and observed under a microscope to confirm whether the specific brain regions had been correctly punched.

Total RNA was extracted from the punched tissues for each rat using TRI reagent (Sigma‐Aldrich, St Louis, MO, USA) in accordance with the manufacturer's instructions. Reverse transcription was then carried out using the reverse transcriptase Superscript II (Invitrogen, Carlsbad, CA, USA) and random primer in accordance with the manufacturer's instructions. Hypoxanthine phosphoribosyltransferase (*HPRT1*) was used as reference gene to normalise the target gene. The PCR primers details are shown in Table 1. The primer pairs selected were designed to amplify across at least one intron, ruling out the possibility of identical size products resulting from genomic DNA amplification. RT‐PCR was then performed using the cDNA obtained via reverse transcription as described above as template. Real‐time quantitative analysis of mRNA levels was performed with the ViiA 7 Real‐Time PCR System (Applied Biosystems, Foster City, CA, USA) using the comparative *C*_T_ (2^−ΔΔCT^) method. During RT‐PCR, the level of amplification of the cDNA samples was made visible in real‐time after each annealing phase via the LightCycler FastStart DNA Master SYBR Green I kit (Roche, Basel Switzerland). For each reaction, 2 μL of sample cDNA (optimised for best PCR amplification results), 0.4 μL each of 10 μmol L^‐1^ antisense and sense primers**,** 0.7 μL of LightCycler FastStart DNA Master SYBR Green I mix, 0.8 μL of MgCl_2_ and 3.7 of μL water were added together to give a total reaction volume of 8 μL. The PCR for CRF, CRF‐R1 and CRF‐R2 was performed under the cycling conditions: Initial denaturation and activation at 95°C for 5 minutes, followed by 40 cycles of denaturation at 95°C for 10 seconds and annealing at 56 and 72°C for 10 seconds. The reaction conditions for *HPRT1* were: 5 minutes at 95°C for one cycle, then 40 cycles of 10 seconds at 95°C, 10 seconds at 56°C and 10 seconds at 72°C. The relative expression of CRF, CRF‐R1 and CRF‐R2 mRNA in control and cat odour groups was determined by the threshold cycle (Ct) value in the exponential phase of the PCR reaction and normalised to the respective *HPRT1* Ct value using the comparative Ct method.^4^

**Table 1:** Sequences of primer pairs for corticotrophin‐releasing hormone (CRF), CRF receptor 1 (CRF‐R1), CRF receptor 2 (CRF‐R2) and hypoxanthine phosphoribosyltransferase 1 (HPRT1) reverse transcriptase‐polymerase chain reaction amplification

	Oligonucleotide primers	Expected size (bp)	Reference
CRF			
Forward	5′‐ACCTGCCAAGGGAGGAGA‐3′	96	NM_031019
Reverse	5′‐GCAGACAGGGCGACAGAG‐3′		
CRF‐R1			
Forward	5‐′TCCACTACATCTGAGACCATTCAGTACA‐3′	248	NM_030999
Reverse	5′‐TCCTGCCACCGGCGCCACCTCTTCCGGA‐3′		
CRF‐R2			
Forward	5′‐TCATCCTCGTGCTCCTCAT‐3′	106	NM_022714
Reverse	5′‐TTCCTGTACTGGATGGTCTCG‐3′		
HPRT1			
Forward	5′‐GCAGACTTTGCTTTCCTTGG‐3′	81	NM_012583
Reverse	5′‐CGAGAGGTCCTTTTCACCAG‐3′		

### 2.3 Evaluation of anxiety‐like behaviour

To determine anxiety‐like behaviour of the predator odour stressed and control rats, the elevated plus maze (EPM) and social interaction tests were performed as described by Poulin et al^24^

#### 2.3.1 Elevated plus maze

The EPM tests were performed at 7‐8 day intervals from PND 21‐43 (four times in total, with the last one after puberty) for the predator odour stressed rats and controls. The EPM consisted of four arms: two facing open arms (51 × 10 cm) and two closed arms (51 × 10 cm), with wall height of 41 cm) and was elevated 72 cm above the floor. Testing was performed under bright white light, which produced approximately 250 lux in the open arms and 170 lux in the closed arms of the EPM. Rats were transferred to the testing room between 9.00 am and 11.00 am and immediately placed in the centre of the maze facing the open arm, and the time spent in open and closed arms was recorded for 5 minutes.

#### 2.3.2 Social interaction

**Figure 1:**
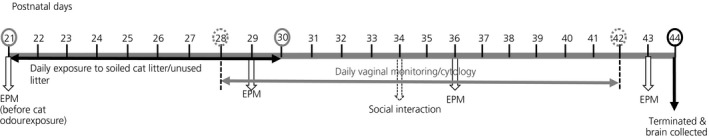
Timeline of the experimental design. Weanling female rats were exposed to soiled cat litter for 10 consecutive days from postnatal day (PND) 21‐30 followed by daily monitoring of vaginal cytology from PND 28‐42 to determine puberty onset and oestrous cyclicity. Rats were tested on the elevated plus maze (EPM) and social interaction was assessed as indicated on the timeline to determine anxiety‐like behaviour. The experiment was terminated on PND 44 and brains were collected for gene expression studies

Before starting the social interaction test, animals were habituated twice (one time per day, 2 days before the day of testing) to the behavioural room for 15 minutes and then to the social interaction arena for 5 minutes on each occasion. The social interaction test was carried out on PND 34, which was 14 days after the commencement of predator odour exposure. Female conspecifics of the same age as the test rats were used for the social interaction test. The time spent on the behavioural events comprising sniffing, chasing, following, grooming and mounting was recorded over a period of 15 minutes by two observers who were blind to the treatment. The tests were carried out between 9.00 am and 11.00 am with the pairs of unfamiliar rats, weight‐matched and placed simultaneously in the arena (50 × 50 × 50 cm). The total social interaction time was calculated by the sum of the time spent in the active social behaviors noted above. A timeline of this experimental design is shown in Figure 1.

### 2.4 MePD osmotic mini‐pump implantation, CRF administration and pubertal timing

All surgeries on the prepubertal rats were carried out under a combination of ketamine (Vetalar, 600 mg/kg, i.p.; Pfizer, New York, NY, USA) and xylazine (Rompun, 60 mg/kg, i.p.; Bayer, Newbury, UK) and were conducted in accordance with the United Kingdom Home Office Regulations.

For this part of the study, a separate group of female rats was chronically implanted unilaterally with 28‐gauge brain cannula (Plastics One Inc., Roanoke, VA, USA) directed towards the MePD on PND 24. Unilateral cannulation was performed to circumvent the technical difficulty associated with bilateral mini‐pump cannulation in small weanling rats. The rats were placed in a stereotaxic frame (Kopf Instruments, Tujunga, CA, USA) and a hole was drilled in the skull at a location above the right MePD after a midline incision was made in the scalp. The coordinates 2.5 mm posterior to bregma (AP), 3.2 mm lateral (ML) and 7.8 mm below the surface of the dura (DV) were determined after carefully optimisation in a group of same age pups in a preliminary experiment and used to target the MePD (AP: −3.3 mm; ML: 3.4 mm; DV: 8.6 mm) in accordance with the rat brain atlas of Paxinos and Watson.^23^ An osmotic mini‐pump (Model 1002; Alza Corp, Mountain View, CA, USA) prefilled with CRF (Sigma‐Aldrich) (n = 12) or artificial cerebrospinal fluid (aCSF) (n = 8) was attached to the cannula with silicone tubing and implanted s.c. in the inter‐scapular space. In PND 24 rats, CRF (0.2 nmol) dissolved in aCSF was delivered at the rate of 6 μL day^‐1^ or aCSF at 6 μL day^‐1^ for a 14‐day period. After the surgical procedure, the rats were allowed to recover on a heated pad until fully conscious. A further group on naïve PND 24 rats (n = 8) served as non‐surgical controls. Vaginal opening was monitored and vaginal smears were taken as described above.

### 2.5 Histological verification of cannula tip position for MePD osmotic mini‐pump implantation

The animals implanted with osmotic mini‐pumps were culled once vaginal oestrus was detected after vaginal opening. The brains were removed, snap frozen on dry ice and stored at −80°C for later histological verification of cannula tip position in brain sections. Brains were cut into coronal sections (thickness of 30 μm) through the MePD region corresponding to bregma −2.80 to −3.60 mm,^23^ mounted on slides and stained with cresyl violet. The infusion site was located from the site of termination of the cannula tract. The boundaries of the MePD and cannula placement were determined using neuroanatomical landmarks with reference guide from the rat brain atlas.^23^ Animals with the cannula outside the MePD were designated as ‘mis‐placed’ and analysed as a separate group in an attempt to determine the specificity of CRF activity to the MePD.

### 2.6 Statistical analysis

Comparisons between control and cat odour‐treated rats with respect to puberty onset (indicated by day of vaginal opening and first oestrus), cycle length, the first EPM trial on PND 21 and social interaction behaviours were made by subjecting data to Student's *t* test, whereas body weight gain and data from the EPM were analysed by two‐way, repeated measures ANOVA with a between‐subjects factor and postnatal day as within‐subject factor, followed by the Holm‐Sidak test where appropriate. Data on the expression of CRF, CRF‐R1 and CRF‐R2 mRNA in the PVN, MeA and CeA tissue samples of control and cat odour‐treated rats were analysed statistically using Student's *t* test. Data on puberty onset in rats unilaterally implanted with mini‐pumps for delivery of CRF or aCSF into the MePD were analysed in comparison with control, aCSF and cannula misplacement groups using a one‐way ANOVA, whereas body weight gain was analysed by two‐way, repeated measures ANOVA with a between‐subjects factor and treatments as within‐subject factor. The results are presented as the mean ± SEM. *P* < 0.05 was considered statistically significant for differences between groups. All statistical analyses were performed using sigmaplot (Systat Software Inc., Chicago, IL, USA).

## 3 RESULTS

### 3.1 Effect of predator odour on puberty onset and body weight

**Figure 2:**
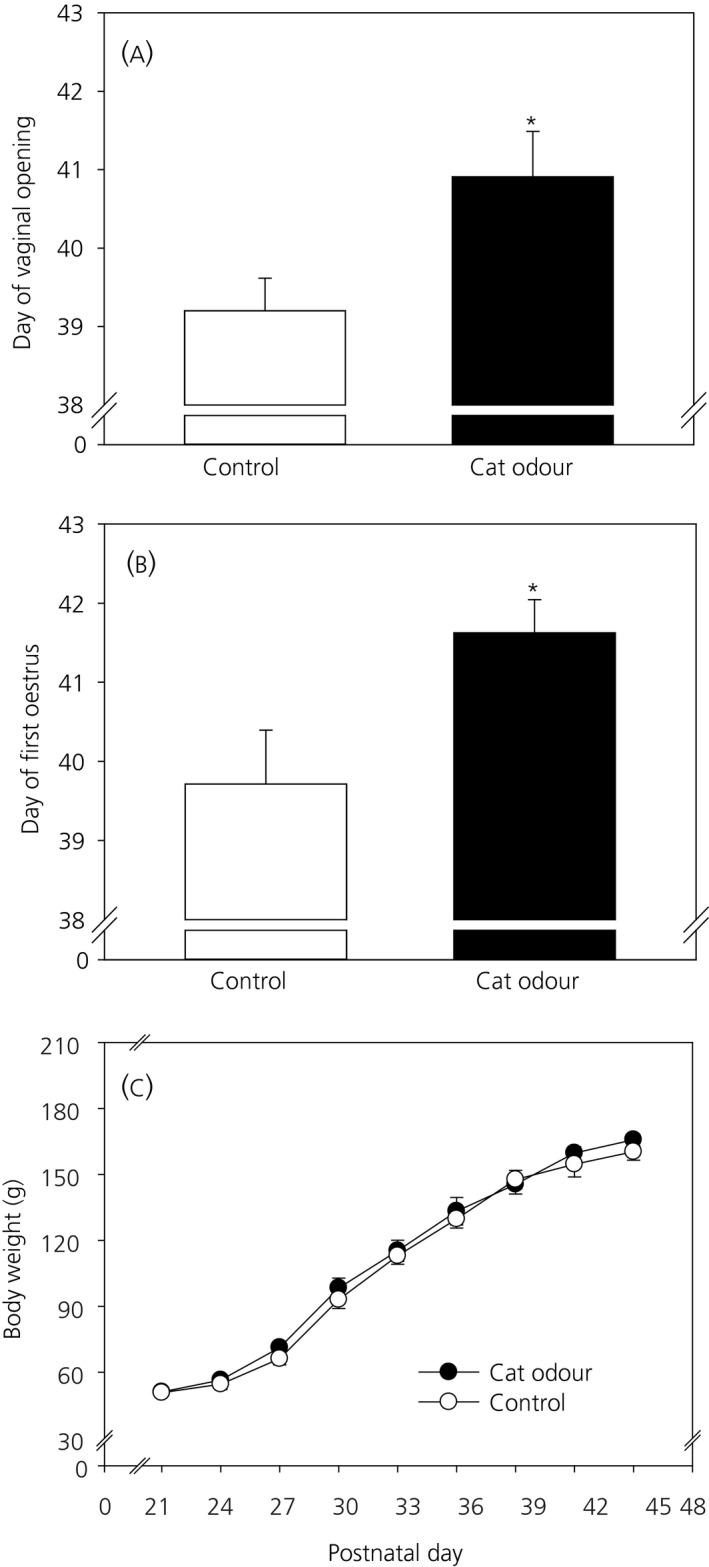
Effect of chronic cat odour stress for 10 days (from postnatal day [PND] 21‐30) on (A) day of vaginal opening, (B) day of first oestrus and (C) body weight gain. Both vaginal opening and first oestrus were significantly delayed in the cat odour‐treated group compared to the controls (*P *<* *0.05). Body weight evolution from PND 21‐45 was not significantly different in control and cat odour‐treated groups analysed by a two‐way, repeated measures ANOVA (*F*_17,378_ = 0.0319, *P *>* *0.05). Data are presented as the mean ± SEM. days after birth. *P <* *0.05 vs control (n = 10‐12 per group)

Chronic cat odour exposure for 10 consecutive days (PND 21‐30) significantly delayed the day of vaginal opening (control: 39.20 ± 0.50 vs cat odour: 40.9 ± 0.50; *P *<* *0.05; n = 10 and 12, respectively) (Figure 2A), as well as day of first oestrus (control: 39.71 ± 0.68 vs cat odour: 41.63 ± 0.42; *P *<* *0.05; n = 10 and 12, respectively) (Figure 2B). A two‐way, repeated measures ANOVA analysis showed weight gain was only affected by the day of postnatal development (*F*_17,378_ = 628.423, *P *<* *0.01) and not by cat odour exposure (*F*_17,378_ = 0.0319, *P *>* *0.05) (Figure 2C).

### 3.2 Effects of predator odour stress on oestrous cyclicity

**Figure 3:**
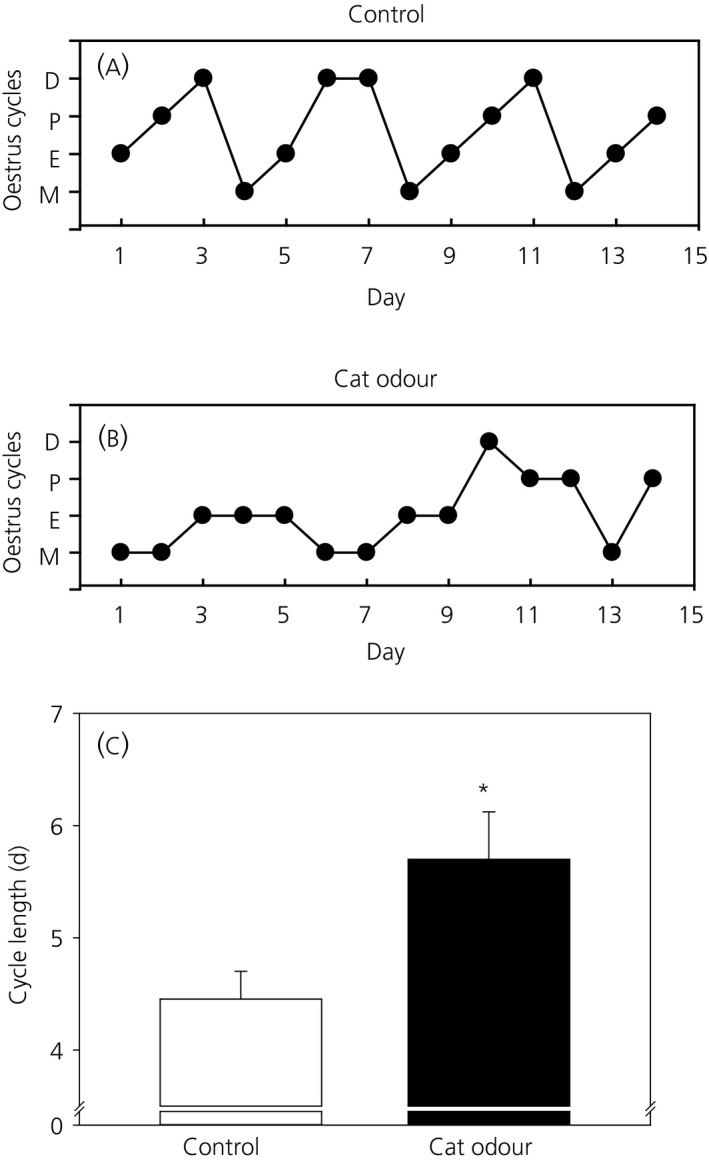
Effect of chronic cat odour stress on oestrous cyclicity and cycle length. Oestrous cyclicity was considered to be normal if at least two consecutive normal cycles, each lasting 4‐5 days including 1‐2 days of oestrus, were observed. Representative oestrous cycles for a control animal (A) shows three normal oestrous cycles with 4‐5 days, and cat odour‐treated animal (B) with abnormal oestrous cycle. D, dioestrus; P, pro‐oestrus; E, oestrus; M, metoestrus. Mean oestrous cycle length (C) was significantly increased in the cat odour‐treated compared to the control group (*P *<* *0.05). Data are presented as the mean ± SEM. *P <* *0.05 vs control (n = 10‐11 per group)

After vaginal opening, oestrous cycle stage was assessed daily for 14 days and the mean oestrous cycle length for control and cat odour groups was calculated. Representative examples of oestrous cycle for control and cat odour‐treated animals are shown in Figure 3A,B. Control animals had a mean oestrous cycle length of 4.4 ± 0.2 days (n = 10) (Figure 3C), which was significantly shorter than the mean oestrous cycle length of 5.7 ± 0.4 days (*P *<* *0.05; n = 11) (Figure 3C) observed in cat odour‐treated animals.

### 3.3 Effect of cat odour stress on CRF and CRF receptor expression in the PVN, CeA and MeA

**Figure 4:**
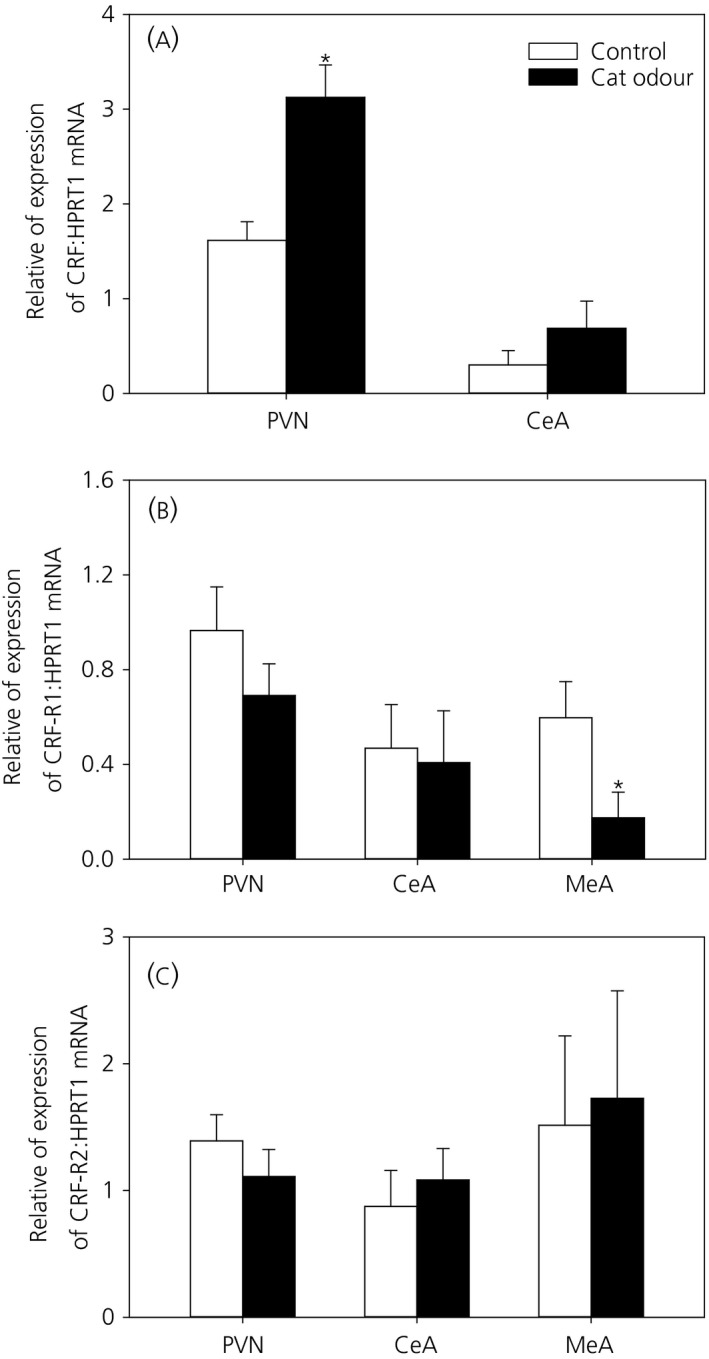
Effect of chronic cat odour stress on corticotrophin‐releasing hormone (CRF), CRF receptor 1 (CRF‐R1) and CRF receptor 2 (CRF‐R2) mRNA expression in the paraventricular nucleus (PVN), as well as the central nucleus of the amygdala (CEA) and the medial nucleus of the amygdala (MEA). Quantification of mRNA levels was carried out using a reverse transcriptase‐polymerase chain reaction from brain micropunch samples of the PVN, CeA and MeA. CRF mRNA levels were significantly higher in the cat odour‐treated group than in the control group in the PVN (*P *<* *0.05) but not in the CeA (A). CRF‐R1 mRNA levels were significantly lower in the MeA of cat odour‐treated rats compared to control rats (*P *<* *0.05), although there were no changes in the PVN or CeA (B). CRF‐R2 mRNA levels in the PVN, CeA or MeA were comparable between the two experimental groups (C). Data are presented as relative levels compared to HPRT1 expression (mean ± SEM). *P <* *0.05 vs control (n = 8‐11 per group)

Relative CRF, CRF‐R1 and CRF‐R2 mRNA levels were measured and compared between control and cat odour‐treated groups in the PVN, CeA and MeA. Cat odour‐treated animals showed a significant increase in relative mRNA expression of CRF in the PVN compared to control animals (*P *<* *0.05; n = 10 for both) (Figure 4A). However, CRF mRNA expression in the CeA was not significantly different in the control and cat odour‐treated groups (*P *>* *0.05; n = 10 and 11, respectively) (Figure 4A). The expression of CRF mRNA in the MeA was not detected and this may be related to the sparse CRF‐immunoreactive cells in the MeA^25^ or the small tissue volume despite pooling samples from both left and right MeA.

Relative CRF‐R1 mRNA levels were not significantly different between control and cat odour‐treated groups in the PVN (*P *>* *0.05; n = 9 for both) (Figure 4B) or CeA (*P *>* *0.05; n = 8 and 11, respectively) (Figure 4B). However, in the MeA, a significant decrease in CRF‐R1 mRNA levels in the cat odour‐treated group can be seen compared to the control group (*P *<* *0.05; n = 11 and 9, respectively) (Figure 4B). Relative levels of CRF‐R2 mRNA were not significantly different in control and cat odour‐treated groups in either the PVN, CeA or MeA (*P *>* *0.05; n = 8‐11) (Figure 4C).

### 3.4 Behavioural effects of chronic predator odour stress

#### 3.4.1 Anxiety‐like behaviour

**Figure 5:**
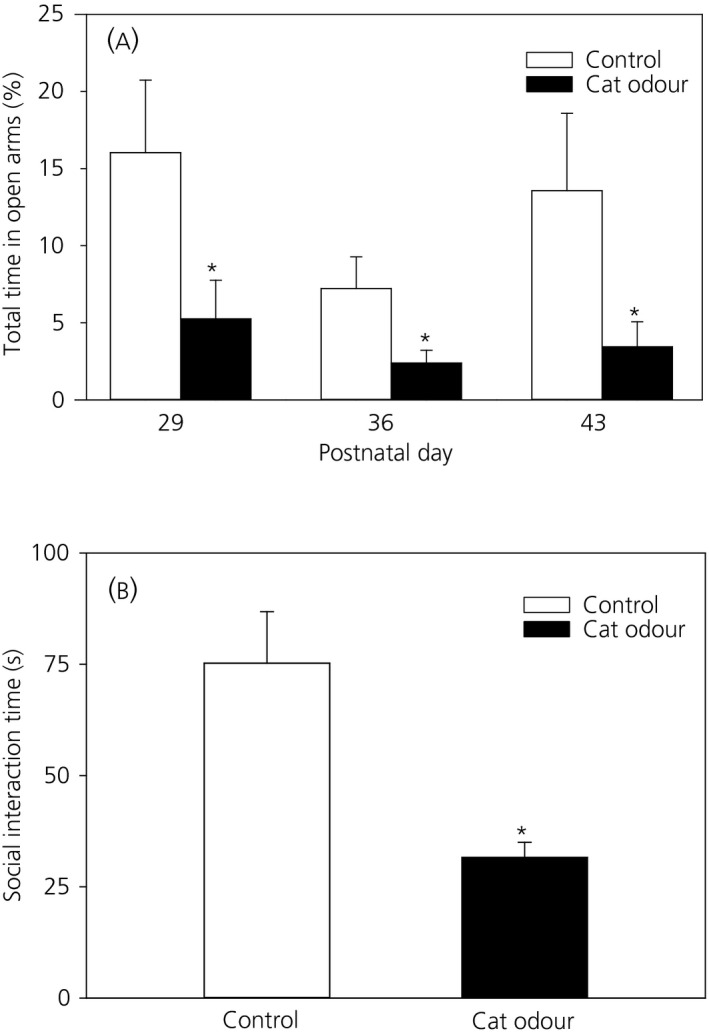
Effect of chronic cat odour stress on phobia/fear‐like behaviour evaluated using the elevated plus maze (EPM) and social interaction test. The activity of the rats on the EPM expressed as percentage of total time spent in the open arms of the EPM was determined on postnatal day (PND) 29, 36 and 43 (A). Across the different test days, there were no significant differences in the time spent by either the control rats or the cat odour treat rats on the EPM open arm (*F*_5,60_ = 3.041, *P *>* *0.05). Comparison between control and cat odour‐treated rats showed that the time spent in the open arms was significantly reduced in the cat odour‐treated group compared to the control group (*F*_5,60_ = 7.370, *P *<* *0.05) and post‐hoc analyses revealed statistical significant on PND 29, 36 and 43 (*P *<* *0.05, on all three test days). Social interaction time (B) was significantly decreased in the cat odour‐treated group compared to the control group (*P *<* *0.05). Data are presented as the mean ± SEM. *P <* *0.05 vs control at the same time point (n = 8‐12 per group)

To assess anxiety and fear‐like behaviour in response to cat odour exposure, standard anxiety and fear tests were performed using the EPM on PND 21 (day of weaning), 29, 36 and 43 (control group: n = 8‐10; cat odour group: n = 10‐12). The first EPM trial on PND 21 confirmed no group difference in anxiety prior to cat odour exposure (total time in open arms; control vs cat odour, 20.76 ± 6.78% vs 24.27 ± 7.49%, *P* > 0.05). It is generally acknowledged that repeat testing in the EPM is assessing a different anxiety construct such as phobia/fear. For the EPM trials on PND 29, 36 and 43, within‐group analysis of behavioural events showed that there were no significant differences in the total time spent in the EPM open arms across the different test days in either control or cat odour‐treated rats (*F*_5,60_ = 3.041, *P* > 0.05) (Figure 5A). By contrast, analysis of anxiety‐like behaviour between the experimental groups (control and cat odour‐treated rats) indicated that cat odour caused a significant decrease in the time spent in EPM open arms (*F*_5,60_ = 7.370, *P *<* *0.05) and post‐hoc analyses revealed statistical significance on PND 29, 36 and 43 compared to the control group (*P *<* *0.05 on all three test days) (Figure 5A), thus providing evidence for an effect of 10 days cat odour exposure (PND 29) and a long‐lasting effect of cat odour (PND 36 and 43) on phobia/fear.

#### 3.4.2 Social interaction

A social interaction test was performed once on PND 34. The time spent in cumulative social interactive behaviour (chasing, sniffing, grooming, following and mounting) was significantly decreased in cat odour‐treated animals (31.6 ± 3.38 s; n = 11) compared to control animals (75.25 ± 11.58 seconds; n = 10; *P *<* *0.05) (Figure 5B).

### 3.5 Effect of intra‐MePD administration of CRF on the timing of puberty

**Figure 6:**
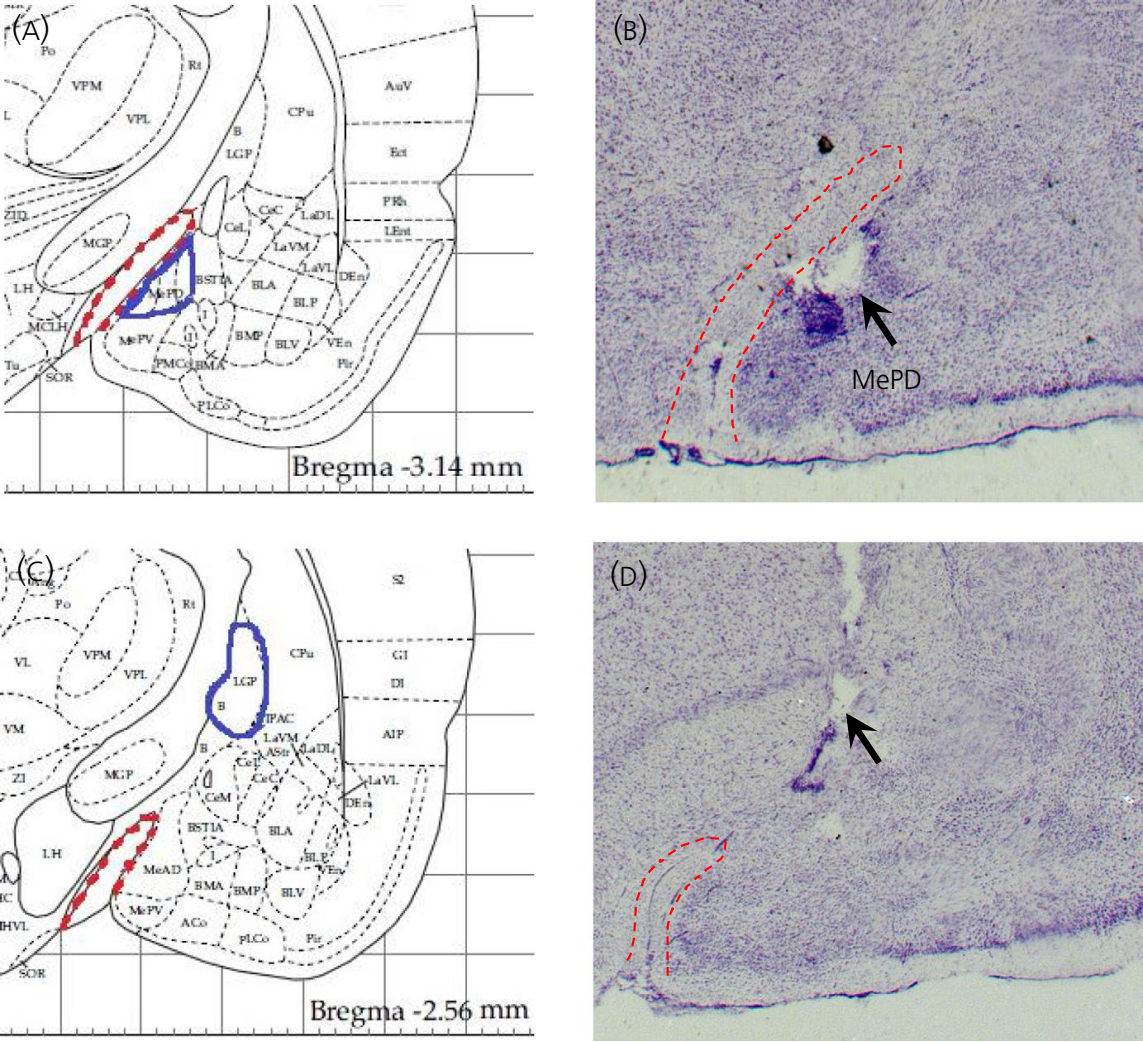
Schematic illustrations and photomicrographs of the osmotic mini‐pump microinfusion sites targeted to the posterodorsal medial amygdala (MePD). Schematic illustration showing the target site for unilateral cannulation of the MePD (A) at bregma ‐3.3 mm, mediolateral ± 3.4 mm and dorsoventral ‐8.6 mm according to the rat brain atlas of Paxinos and Watson.^23^ The approximate border of the MePD (blue line) with its spatial relationship with the optic tract (red dotted line) is shown in the schematic drawing. Photomicrograph of a coronal brain section in a representative animal implanted with the cannula in the MePD for infusion of corticotrophin‐releasing hormone (0.2 nmol in 6 μL day^‐1^) or artificial cerebrospinal fluid (6 μL day^‐1^) for 14 days. An arrow indicates the site corresponding to the tip of the cannula (B). Schematic drawing illustrating the site of infusion outside of the target area (MePD) at bregma (AP) ‐2.56 mm according to the rat brain atlas of Paxinos and Watson.^23^ The blue line bordered the globus pallidus**,** whereas the red dotted line indicates the approximate border of the optic tract (C). A representative photomicrograph of coronal brain section of an animal implanted with the cannula misplaced outside of the target area (MePD). An arrow indicates the site corresponding to the tip of the cannula (D)

**Figure 7:**
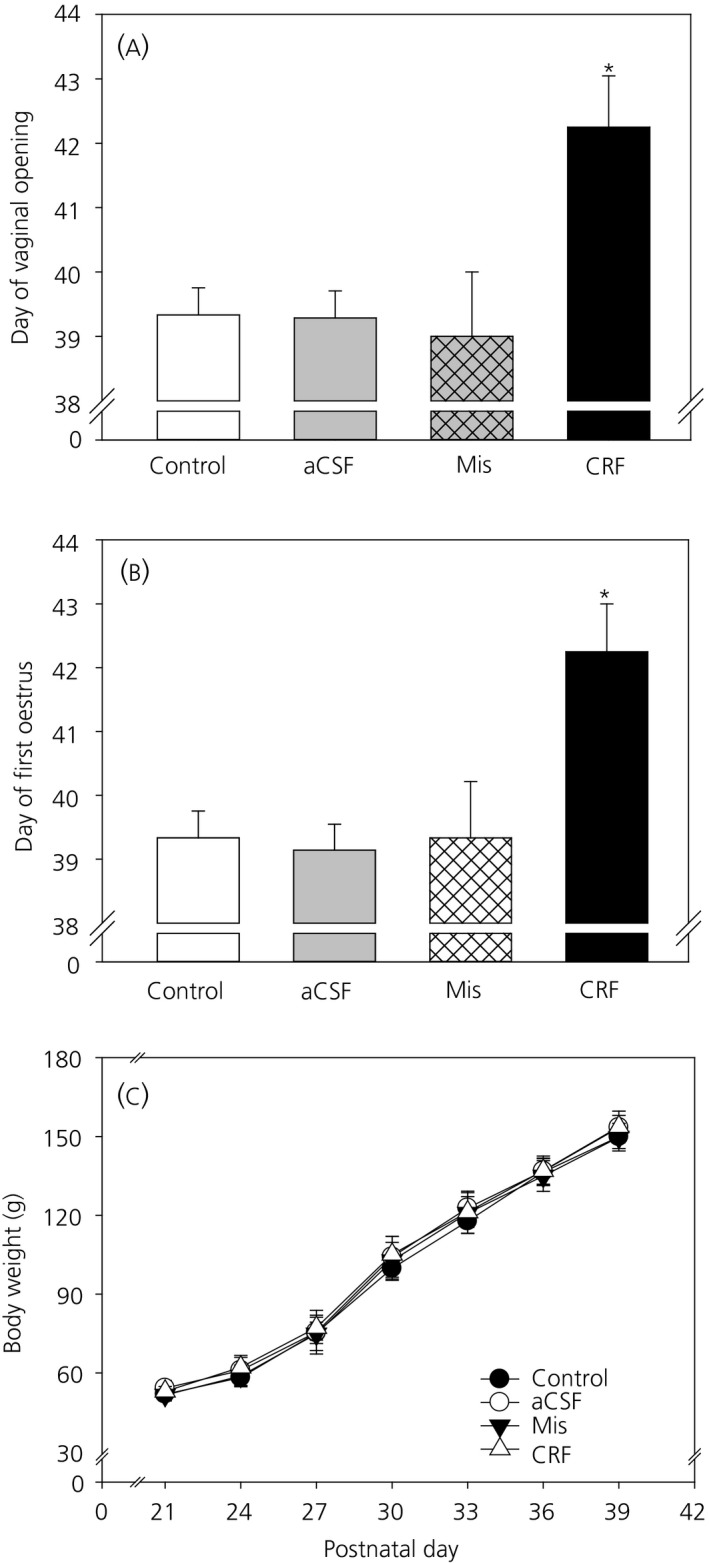
Effect of intra‐posterodorsal medial amygdala (MePD) administered corticotrophin‐releasing hormone (CRF) on the timing of puberty and body weight change. Chronic infusion of CRF (0.2 nmol day^‐1^) via an osmotic mini‐pump for 14 days starting from postnatal day 24 into the MePD significantly delayed the day of vaginal opening (*F*_3,20_ = 5.927, *P *=* *0.005) (A) and first oestrus (*F*_3,20_ = 6.637, *P *<* *0.05) (B) compared to non‐surgical (*P *<* *0.05) and artificial cerebrospinal fluid (aCSF) control groups (*P *<* *0.05), as well as the misplaced cannulae (Mis) group (*P *<* *0.05). Chronic intra‐MePD infusion of CRF had no significant effects on the body weight gain (C) compared to the control groups and misplaced cannulae group (*F*_3,576_ = 0.280, *P *>* *0.05). Data are presented as the mean ± SEM. *P <* *0.05 vs control groups (n = 7‐9 per group). Misplaced cannula group (n = 3)

Cannulae placement in the MePD was verified in coronal brain sections. Of the 12 rats that received CRF, three had cannulae that were not located in the MePD (Figure 6). These animals with misplaced cannulae were separately analysed as the “misplaced” group. A one‐way ANOVA analyses showed that chronic administration of CRF (0.2 nmol, 6 μL day^‐1^) in the MePD delayed the timing of puberty onset, as marked by an approximate 3‐day delay in vaginal opening compared to the control groups and the misplaced cannula group (*F*_3,20_ = 5.927, *P *=* *0.005) and post‐hoc analysis showed a statistically significantly difference between CRF treatment mice and all other experimental groups (vaginal opening: non‐surgical control, 39.3 ± 0.42 days; aCSF, 39.2 ± 0.42 days; misplaced‐CRF, 39.0 ± 1.00 days; CRF; 42.3 ± 0.80 days; *P *<* *0.05; n = 7‐9 per group, except for the misplaced cannula group n = 3) (Figure 7A). A similar trend was observed for the day of first oestrus. The animals that received CRF in the MePD had a significant delay in the day of first oestrus compared to non‐surgical and aCSF‐treated control groups, as well as the misplaced cannula group (*F*_3,20_ = 6.637, *P *<* *0.05) (first oestrus: control, 39.7 ± 0.49 days; aCSF, 39.6 ± 0.37 days; misplaced‐CRF, 39.7 ± 0.88 days; CRF, 42.8 ± 0.77 days; *P *<* *0.05; n = 7‐9 per group, except for the misplaced cannula group n = 3) (Figure 7B). Furthermore, measurement of the body weight gain showed no significant variations between the experimental groups (*F*_3,576_ = 0.28, *P *>* *0.05) (Figure 7C).

## 4 DISCUSSION

The results of the present study demonstrate that chronic exposure to cat odour delays puberty onset, disrupts oestrous cyclicity and elicits fear responses in female Sprague‐Dawley rats. These phenotypic and behavioural outcomes were accompanied by increased CRF expression in the PVN and decreased CRF‐R1 mRNA levels in the MeA. Furthermore, intra‐MePD administration of CRF mimics the same changes to pubertal timing and oestrous cyclicity as those elicited by predator odour exposure.

Predator odour stress is a model for PTSD, causing physiological and behavioural changes similar to those seen in patients with the disorder, including increased anxiety and fear, as well as decreased social interaction.^26^ Predator stress also induces synaptic plasticity in brain regions involved in fear and defensive behaviour such as the amygdala.^27^ In the present study, the manifestation of increased fear and decreased sociability in rats exposed to predator stress is consistent with earlier reports in rats and corroborates key features of PTSD.^28^ The timing of trauma exposure relative to puberty has been shown to confer increased vulnerability to anxiety in humans,^29^ which is supported by evidence of increased stress‐induced c‐Fos expression in CRF cells of the PVN in pre‐pubertal compared to adult rats.^30^ Furthermore, our data demonstrate an inverse relationship between anxiogenic behaviour and female sexual maturation. The manifestation of delayed puberty in the cat odour‐treated rats may be related to synaptic plasticity of key brain regions as a result of stress exposure 31 that impinge on the neural circuits controlling pubertal development.

The amygdala mediates fear and defensive behaviour in rodents in response to predator odour.^27^ The MeA is the first site of convergence of olfactory and vomeronasal information^32^ and exhibits a robust response to cat odour.^12^ However, it is not clear how predator odour signals activate the HPA axis. In response to psychological stress, the MeA is considered to exert an activating influence on HPA function,^33^ which is supported by attenuation of stress‐induced PVN activation and adrenocorticotrophic hormone (ACTH) secretion in rats with MeA lesions.^34^ The vast majority of neural projections from the MeA to the PVN are GABAergic, which are assumed to promote CRF release via disinhibition of GABAergic inter‐neurones directly presynaptic to the CRF cell populations in the PVN.^8^ Specifically, chemogenetic activation of MeA neurones has been shown to increase both c‐Fos expression in CRF PVN neurones and plasma levels of ACTH.^35^ It is plausible that the excitatory effect of predator odour on the MeA^12^ promotes HPA activation by increased CRF expression in the PVN, as observed in the present study. Indeed, up‐regulation of CRF mRNA expression in the PVN is a common outcome in adult rats exposed to cat odour and may occur without any corresponding change to corticosterone levels,^36^ implying a lack of feedback signal to control HPA output. The lack of effect of cat odour on CRF or CRF receptor expression in the CeA is commensurate with the minimal response of the CeA to predator odour.^12^ Indeed, markers of neuronal activation were unchanged in the CeA of rats exposed to predator odour^37^ and CeA lesions did not alter cat odour‐induced fear responses,^38^ indicating that the CeA may not be critically involved in coordinating neuroendocrine responses to predator odour. However, with restraint stress, a rapid and sustained CRF release in the CeA has been reported.^33^

CRF‐R1 and CRF‐R2 were unchanged in the PVN and CeA, although, in the MeA, CRF‐R1 was significantly down‐regulated in rats exposed to cat odour, which may be suggestive of an enhanced CRF signalling in the MeA. CRF is known to down‐regulate CRF‐R1 expression in the prefrontal cortex in response to unpredictable stress.^39^ Similarly, decreased CRF‐R1 expression has been observed in the frontal cortex of suicide victims suffering from depression,^40^ potentially caused by chronic elevated levels of CRF in the CSF.^41^ In the present study, the origin of the potential CRF inputs to the MeA are unknown. Although we observed a lack of change in CRF expression in the CeA of the cat odour‐exposed rats, this may reflect a situation where the endogenous CRF released in the amygdala has become habituated to chronic stress as a protective mechanism against CRF over‐expression but, nevertheless, this is sufficient to yield a down‐regulation of MeA CRF‐R1 expression, potentially from CeA afferent projections to the MeA.^42^ It had been shown that up‐regulation of CRF expression by a lentiviral vector specifically in the CeA changed puberty onset in female rats, although this comprised an advancement.^2^ However, the CRF expression in the CeA in the present study is not increased in the cat odour‐exposed rats, at least at the time of tissue collection. The timing of puberty onset may be advanced by high levels of CRF expression in the CeA but delayed by CRF signalling in the MePD. Although neural projections from the PVN to the MeA have been demonstrated in sheep,^43^ they are rarely seen in rodents,^42^ which tempers the possibility of a direct CRF input from the PVN to down‐regulate MeA CRF‐R1 expression. Activation of CRF‐R1 in the MeA could be mediated by the urocortins. However, the only urocortin that is highly expressed in the MeA and involved in behavioural responses to stress is urocortin‐3, which selectively activates CRF‐R2 and not CRF‐R1.^44^ Further studies are required to establish the source of CRF input onto the MeA.

The phenotypic sign of delayed puberty as a result of chronic cat odour exposure may be related to changes in the neural circuits that govern puberty timing vis‐à‐vis the influence of the stress neuropeptide, CRF, which negatively influences the HPG axis.^45^ Of note, the MeA plays an important role in puberty and its MePD subnucleus is particularly involved in regulating puberty timing.^18^ Neural activity within the MeA between PND 20 and 30 is crucial with respect to determining the timing of puberty, with increased MeA activity being related to delayed puberty onset.^46^ The female rats in the present study were exposed to cat odour during this critical window and, because cat odour increases neuronal activity in the MeA,^12^ this suggests that changes in MeA activity may underlie the puberty delay in response to predator stress. The down‐regulation of CRF‐R1 expression in the MeA would suggest an involvement of CRF signalling in the MeA mediating the pubertal delay. The potential role of CRF signalling in bridging the stress response in the MeA with sexual maturation was furthered by our observation that chronic administration of CRF specifically into the MePD delayed puberty onset in female rats. Thus, CRF signalling specifically in the MePD is critical for timing of puberty, and further supports the important role played by this amygdaloid nucleus in controlling puberty onset.^18^

The disruption in oestrous cyclicity observed in the present study agrees with previous studies using chronic mild stress 47 and may be attributed to the enhanced CRF expression in the PVN, although this contrasts with the expectation of a minimal role for PVN CRF in stress‐induced reproductive dysfunction.^5^ Nevertheless, CRF exerts a suppressive effect on GnRH/LH secretion^48^ that may involve GABAergic inputs to GnRH neurones.^49^ Over‐expression of CRF in the CeA, comprising a model of chronic stress in rats, resulted in irregular oestrous cyclicity, as well as suppression of the preovulatory LH surge,^2^ although the mechanisms underlying ovulatory dysfunction are unclear because there are few projections from the CeA to the hypothalamic reproductive nuclei controlling ovulation.^50^ Stressful stimuli may also recruit the MeA to suppress the HPG axis.^11^ The presence of CRF receptors in the MeA 51 and direct synaptic connection between MeA GABAergic neurones and GnRH neurones in the mPOA^52^ might suggest that CRF signalling in the MeA could underlie the modulatory effect of the MeA on the ovulatory processes^18^ by disrupting the GnRH neural network. Whether the long‐term impact of exposure to cat odour on ovarian cyclicity is mediated by similar mechanisms remains to be established.

The results of the EPN and social interaction tests demonstrate the severity of cat‐odour exposure on anxiety‐like behaviour in female rats. CRF‐R1 activation is associated with the initiation and consolidation of the long‐term effects on anxiety‐like behaviours caused by exposure to predator stress,^27^ whereas CRF‐R2 is more commonly related to modulating the recovery response.^53^ Although CRF‐R2 mRNA expression was unchanged in the brain nuclei examined, which may be part of a ‘stress coping’ mechanism, the reduced expression of CRF‐R1 in the MeA may underlie a homeostatic mechanism to curtail an enhanced fear response that could be damaging to physiological processes. Remarkably, earlier studies have shown that MeA lesions attenuate anxiety‐like behaviour after predator stress.^54^ Therefore, CRF‐R1 activity in the MeA may be implicated in predator exposure‐induced fear in female rats to support a synchrony between anxiety‐like behaviour and altered reproductive function under stressful conditions.

A limitation of the present study was the absence of using neuropharmacological CRF receptor antagonism to reverse the negative effect of cat odour exposure on pubertal timing. Given the technical challenge of the required surgical manipulation in small animals, our planned future experiments involving a chemogenetic approach should successfully address this limitation. In addition, the small size of the punched brain tissues hampered the possibility of a separate confirmatory assay to determine protein levels of CRF and its receptors in the rats exposed to cat odour, as well as those infused with CRF, which would have strengthened the present data set.

In conclusion, the results obtained in the present study suggest a direct involvement of CRF signalling specifically in the MePD in puberty timing that may be critical for predator odour‐induced pubertal delay.

## CONFLICT OF INTERESTS

The authors declare that they have no conflicts of interest.
